# The Effectiveness of a Nonguided Mindfulness App on Perceived Stress in a Nonclinical Dutch Population: Randomized Controlled Trial

**DOI:** 10.2196/32123

**Published:** 2022-03-18

**Authors:** Leonieke W Kranenburg, Jamie Gillis, Birgit Mayer, Witte J G Hoogendijk

**Affiliations:** 1 Section of Medical Psychology Department of Psychiatry Erasmus University Medical Center Rotterdam Netherlands; 2 Department of Psychology, Education and Child studies Erasmus University Rotterdam Rotterdam Netherlands; 3 Department of Psychiatry Erasmus University Medical Center Rotterdam Netherlands

**Keywords:** mHealth, mindfulness, stress, burnout, non-clinical population, nonclinical

## Abstract

**Background:**

Mindfulness has become increasingly popular, and positive outcomes have been reported for mindfulness-based interventions (MBIs) in reducing stress. These findings make room for innovative perspectives on how MBIs could be applied, for instance through mobile health (mHealth).

**Objective:**

The aim of this study is to investigate whether a nonguided mindfulness mobile app can decrease perceived stress in a nonclinical Dutch population over the course of 8 weeks, with follow-up at 6 months.

**Methods:**

A randomized controlled trial was performed to compare an experimental group that made use of a structured 8-week mHealth mindfulness program and a control group after 8 weeks, with follow-up after 6 months. Participants were recruited via a national television program. The primary outcome measure was perceived stress as measured by the Perceived Stress Scale, secondary outcomes were symptoms of burnout (measured using the visual analog scale [VAS]) and psychological symptoms (measured using the Four-Dimensional Symptom Questionnaire [4DSQ] at follow-up). Outcomes were analyzed using a multilevel regression model.

**Results:**

At baseline, 587 respondents were included. Results showed no postintervention differences between groups for the level of perceived stress. With regard to the secondary outcome measures, the VAS for *emotional exhaustion* and *physical exhaustion* showed significantly lower scores for the experimental group after 8 weeks (*P*=.04 and *P*=.01, respectively), but not at follow-up. There were no differences between groups for psychological symptoms measured using the 4DSQ.

**Conclusions:**

These findings do not support our hypothesis that using the mindfulness app would reduce stress levels. However, our findings related to diminished exhaustion at 8 weeks are encouraging and require further investigation.

**Trial Registration:**

ClinicalTrials.gov NCT05246800; https://clinicaltrials.gov/show/NCT05246800

## Introduction

Mindfulness practice has become increasingly popular, both in the general public [1,2] and in health care [3]. Mindfulness derives from Buddhism and can be defined as an ability to observe one’s bodily sensations, feelings, and thoughts with an open, nonjudgmental, and accepting mind toward one’s experiences [4]. Mindfulness has been found to alleviate intense emotional states [5,6] and enhance emotional coping mechanisms in response to stress [7], and it is believed to induce shifts in processing of negative emotions under stress [8]. The core principles of mindfulness are incorporated in a variety of psychological treatments that are referred to as mindfulness-based interventions (MBIs).

MBIs are traditionally delivered face to face. New types of applications include web-based programs and mobile health (mHealth) [9]. Advantages of digital applications of MBIs include their availability and accessibility, avoiding waiting lists, saving travelling time, reduced costs, being able to work in one’s own environment, and nonrequirement of a therapist [10,11]. Especially during the current COVID-19 outbreak, these are favorable assets. Reviews of studies on the efficacy of these web-based MBIs found up to moderate effects on stress and depression [7,12-16]. A recent meta-analysis on the efficacy of mindfulness meditation apps on users’ well-being and mental health–related outcomes concluded that mindfulness apps seemed promising in improving well-being and mental health, but those results should be interpreted with caution [17]. The strongest effects were observed on stress, depression, and burnout. However, regarding burnout, only 3 studies could be included, indicating that this may be a relatively new outcome variable in this research field. The findings of Gál et al [17] are interesting in this respect as chronic stress, burnout, and depression can be viewed as a continuum. Chronic stress can lead to burnout and a great overlap exists between burnout and depression, with shared features including motivational problems and exhaustion [18,19]. In total, 17% of all employees in the Netherlands report burnout symptoms [20], and 5% of all Dutch adults are annually affected by depression [21]. During the COVID-19 pandemic, these numbers may even be higher, as reports worldwide point to increased mental health complaints for various population subgroups, such as health care professionals, teachers, and those working from home [22-25]. The strain on mental health care budgets and practices, characterized by long waiting lists and shortness of qualified personnel, make it even more important to invest in new technologies to reduce mental health problems in the general population [22]. Therefore, the aim of this study is to investigate whether a nonguided mindfulness mobile app can decrease perceived stress levels and burnout symptoms in a nonclinical Dutch population.

## Methods

### Design and Recruitment

A randomized controlled trial with follow-up measures at 6 months was performed. Participants were recruited through the television (TV) program *Kassa*, in the context of 4 broadcasts on the topic “stress” in March 2018. The study was announced during this TV show, and in that particular moment, viewers were invited to visit the program’s website and click the weblink with more information about the study and the possibility to apply for it. There were no eligibility criteria, other than being an adult. After having read and accepted the terms and conditions (informed consent), participants were referred to the web-based questionnaires. Randomization took place after they had filled out the baseline (T0) questionnaires and was performed with a built-in randomization algorithm. The experimental group was provided access to the mindfulness mobile app, which contained an 8-week nonguided mindfulness program developed to reduce stress symptoms. The control group was suggested to read information about stress and burnout on the *Kassa* website. Participants were not blinded to their condition, as they knew whether or not they received access to the mindfulness app. There were three time points of measurement: T0 (baseline, before randomization), T1 (at the end of the program, 8 weeks after randomization), and T2 (6 months after randomization).

### Intervention

The mindfulness application was developed by Minddistrict, an eHealth company in the Netherlands. The content of the app was developed by professionals in the field of mental health care and in accordance with the principles of mindfulness-based stress reduction and mindfulness-based cognitive therapy [4,26]. This mindfulness mobile app was the first app version derived from the one already existing web-based mindfulness program at the time used in mental health care settings. The app consisted of a structured program, with chapters on psycho-education on mindfulness and the importance of practicing; acting on auto-pilot, conscious attention; nonjudgmental attention, awareness; doing versus being mode; attention for breath and body, conscious response; acceptance; a mindful attitude toward thoughts; and applying mindfulness in daily life and staying mindful. Each chapter started with a short explanation of a specific mindfulness principle and was followed by relevant exercises, such as the body scan, Raisin Exercise, breath exercises, and sitting meditation. After completion of the exercises, participants were asked about their experiences, and the participant received an encouraging standard feedback message to keep practicing the exercises for optimal mindfulness training. There was no real-life contact (either in person or on the internet) with a mindfulness trainer. There was the possibility to create a personal program with favorite exercises.

### Measures

#### Demographics

Participants age, sex, level of education, and occupation were assessed.

#### Perceived Stress Scale

The PSS measures perceived stress levels [27]. The 14-item Dutch version was used in this study. All items are rated on a 4-point Likert scale, with higher scores indicating more perceived stress. Cronbach α ranges between .84 and .86 [27] and overall psychometric properties are evaluated as acceptable [28]. The Cronbach α for this sample was >.89 for all measurement time points.

#### Visual Analogue Scale

Burnout symptoms were assessed using 8 visual analogue scales (VASs). The symptoms measured were as follows: *control over emotions*, *memory and concentration*, *sleep*, *work interest*, *work performance*, *interest in others*, *emotional exhaustion*, *and physical exhaustion*. Each symptom was rated on a 0-100 scale, with higher scores indicating higher difficulty.

#### Four-Dimensional Symptom Questionnaire

The Four-Dimensional Symptom Questionnaire (4DSQ) consists of 50 items rated on a 4-point Likert scale [29]. The 50 items can be grouped into four dimensions: Distress (n=16), Depression (n=6), Anxiety (n=12), and Somatization (n=16). Sum scores are calculated for each dimension. The reliability of these dimensions was good, with Cronbach α>.79 for all subscales [30]. The Cronbach α for this sample was >.88 for all subscales. This measure was applied at T2 only.

### Statistical Analyses

The experimental group and the control group were compared using a multilevel regression analysis, with participants as the upper level and their repeated measures as the lower level. Time (repeated measures at T0, T1, and T2), treatment group (experimental or control), and the time–group interaction were postulated as fixed effects. The difference in change in the PSS and VAS between the groups at follow-up is considered the primary contrast. At T2, differences between groups for the 4DSQ were analyzed with an independent samples *t* test (2-tailed). Two-sided *P* values of <.05 were considered significant. Data were analyzed using descriptive statistics in SPSS (version 25; IBM Corp).

### Ethical Considerations

The study was approved by the Erasmus University Medical Ethical Committee and evaluated as not subject to the Dutch act on medical scientific research involving human subjects (METC 2017-1117).

## Results

### Participants

**Figure 1:**
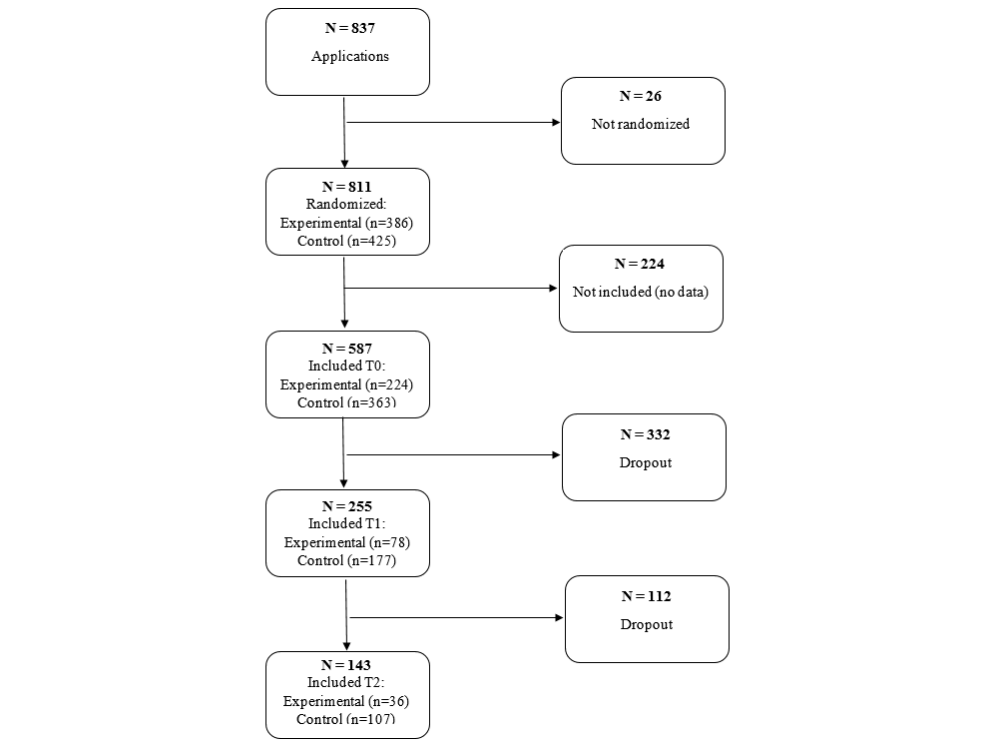
Flowchart of inclusion and dropout.

The final sample at T0 included 587 participants. This sample consisted mostly of highly educated (64.5%), employed (74.7%), and female (74.6%) individuals with a mean age of 46.05 (SD 13.64) years. More detailed information about the initial and final sample is provided in Table 1. Figure 1 provides the participant randomization flowchart.

**Table 1:** Demographic variables.

Variables			Time points		
			T0		T2
Age (years), mean (SD)			45.86 (13.69)		47.70 (12.80)
**Sex, n (%)**					
	Female	435 (74.1)		137 (74.8)	
	Male	152 (25.9)		36 (25.2)	
**Education, n (%)**					
	Elementary school	2 (0.3)		1 (0.7)	
	Middle and high school	72 (12.3)		14 (9.8)	
	Secondary education	129 (22.0)		34 (23.8)	
	Higher education	384 (65.4)		94 (65.7)	
**Employment status, n (%)**					
	Social welfare	7 (1.1)		3 (2.1)	
	Informal care	9 (1.5)		0 (0)	
	Unemployed	14 (2.4)		6 (4.2)	
	Volunteers	21 (3.6)		5 (3.5)	
	Domestic household	21 (3.6)		4 (2.8)	
	Retired	34 (5.8)		11 (7.7)	
	Sick leave	38 (6.5)		13 (9.1)	
	Part-time	206 (35.0)		47 (32.9)	
	Full-time	237 (40.0)		54 (37.8)	

### Primary Outcomes

Perceived stress was measured with the PSS at T0, T1, and T2. Table 2 provides descriptive statistics of the PSS over time for both groups. Changes between groups over time were analyzed with a multilevel regression model. The interaction between group and time was not a significant predictor of perceived stress measured using the PSS at T1 (*F*_1,501.26_=0.70, *P*=.40) and T2 (*F*_1,490.35_=1.36, *P*=.24; Table 3).

**Table 2:** Descriptive statistics of the perceived stress scale (PSS).

Group		PSS score, mean (SD)	Participants, n
**T0**			
	Control	29.33 (8.32)	363
	Experimental	30.10 (7.67)	224
**T1**			
	Control	27.51 (9.21)	174
	Experimental	26.59 (8.83)	73
**T2**			
	Control	26.30 (9.98)	107
	Experimental	26.00 (9.72)	36

**Table 3:** Estimates of the fixed effects measures of the perceived stress scale (PSS; dependent variable: PSS score).

Interaction	Estimate (95% CI); SE	*P* value
Group×T1	–0.71 (–2.38 to 0.96); 0.85	.40
Group×T2	–1.29 (–3.47 to 0.89); 1.11	.24

### Secondary Outcomes

There were 8 VASs measuring burnout symptoms at T0, T1, and T2. Changes between groups over time were measured with a multilevel regression model. The VAS scales of *emotional exhaustion* and *physical exhaustion* were both significant at T1 (*F*_1,520.41_=4.16, *P*=.04 and *F*_1,528.76_=6.29, *P*=.01, respectively), for the time–group interaction, with the experimental group presenting lower exhaustion scores. Upon 6-month follow-up (T2), this effect was not maintained with the outcomes (*F*_1,510.18_=0.03, *P*=.87 and *F*_1,520.06_=0.04, *P*=.84, respectively). Changes between the two groups over time for the other 6 VAS scales did not differ at T1 and T2. Outcomes for the 4DSQ subscales at T2 showed no significant differences between both groups.

## Discussion

### Principal Findings

Our primary research question served to investigate whether an 8-week nonguided mindfulness mobile app can decrease perceived stress levels in a nonclinical Dutch population. Our findings do not support our hypothesis that using a nonguided mindfulness app reduces perceived stress levels. Not observing an effect on stress in this study might be explained by the complete lack of personal contact in this study; that is, there was no mindfulness trainer reachable through the app, nor was there the possibility to engage in social contact with other participants. This potential hypothesis is supported by the recent review of Borghouts [31], which points toward a generally higher engagement for guided (vs unguided) interventions and to the importance of social connectedness as a facilitator of user engagement. In addition, a recent meta-analysis [32] focusing specifically on web-based mindfulness found that web-based MBIs resulted in higher effect sizes for stress when offered guidance. As there was also no personal contact between the research team and the respondents, one could say that our study results may be similar to what one might expect to find in a real-world user situation, where despite high levels of app download, only a small portion of users actually use the apps for a longer period [33,34].

Another finding of our study was that the experimental group reported lower levels of both emotional and physical exhaustion after 8 weeks of using the app. This finding is particularly interesting, as different definitions of burnout all share exhaustion as a central component [35-38]. For instance, according to Schaufeli et al [38], burnout is conceptualized as a state of mental exhaustion, leading to both an inability and an unwillingness to act. Furthermore, in the process model of burnout, emotional exhaustion is one of the first symptoms to develop [39]. Dealing with stressors in everyday life can result in the depletion of cognitive and emotional resources, and these can cause exhaustion [40-42]. It is striking that the possible gain of the app may lie in reducing feelings of exhaustion in the broad sense. This might be related to the role mindfulness plays in autonomous self-regulation [43], which preserves vitality and energy [44]. Hence, a better spending and preservation of cognitive and emotional resources through increased self-regulation might have resulted in a reduction of physical and emotional exhaustion after 8 weeks. This finding is in line with other studies that reported that mindfulness interventions are related to a reduction of emotional exhaustion, both in health care professionals [45,46] and other employees [47]. However, when it comes to mobile mindfulness apps, to our best knowledge, only one previous study specifically reported on exhaustion outcomes. In this study, significant effects were found for reduced emotional exhaustion [48]. These findings might indicate that a mindfulness app has the potential to be used as preventive intervention for burnout in a nonclinical population.

### Clinical Implications and Directions for Future Research

Future research on the effects of mobile mindfulness apps on burnout is warranted. Given the long waiting lists for mental health care, an ideal setting for further testing this or other mobile mindfulness apps would be general practitioner clinics and mental health care institutions. That is, driven by these long waiting lists, it has become more common to offer patients bridge interventions with low personnel costs, including eHealth modules. This provides a great opportunity to implement a study design with conditions that vary in the amount and type of personal contact. Such a design could even be advanced by using randomization schemes that allow for including patient preferences with regard to these aspects. Next, given our findings that both mental and physical exhaustion decreased in the app user group, it would be of great interest to conduct frequent measures of individual stress and burnout symptoms with an experience sampling method [49]. This can help build general networks of how burnout symptoms develop and worsen over time [50]. By drawing on such data, future app-based interventions could be personalized to increase effectiveness. Building a user-friendly experience sampling method incorporated in the mindfulness app would then be the next challenge for app developers. In addition, build-in measures for actual time spent on practicing mindfulness exercises would also contribute to the field, as until now such data are limited [1]. Of course, such new features should first be subjected to acceptability and feasibility studies.

### Limitations

The fact that participants were unequally distributed between the experimental and the control group was a limitation of this study. This was owing to a previously unnoticed error in the automated randomization algorithm, which could unfortunately not be repaired afterward. Another limitation is the lack of an adherence measure, which makes it impossible to look at a possible dose-response relationship [1]. Furthermore, the open kind of recruitment via a TV program may have biased our sample. For instance, it could be that extravert persons were more likely to spontaneously act upon the invitation to visit the website and register for the study. As extraversion is a barrier for user engagement of digital mental health interventions [31], the way participants were recruited could have biased our sample. This of course is only a hypothesis, as we have no data on personality traits of our sample, or on other relevant user characteristics, such as mental health status and previous experiences with mindfulness or meditation, which may have influenced both user engagement and the outcomes.

### Conclusions

Our study did not find an effect of using a mindfulness app on perceived stress levels in a nonclinical Dutch population. However, this may be owing to the type of respondent recruitment and a lack of control on adherence levels. Our results show diminished emotional and physical exhaustion in the app user group after 8 weeks. These findings are encouraging as they suggest that a mindfulness app has potential to be used as a preventive intervention for burnout.

## Appendix

Multimedia Appendix 1 CONSORT-eHEALTH checklist (V 1.6.1).

## Abbreviations

4DSQ: Four-Dimensional Symptom Questionnaire
MBI: mindfulness-based interventions
mHealth: mobile health
PSS: perceived stress scale
TV: television
VAS: visual analogue scale
